# A novel case of renal pathergy reaction in a Behçet’s disease patient complicated by IgA vasculitis

**DOI:** 10.1186/s12882-017-0451-7

**Published:** 2017-01-28

**Authors:** Takaaki Higashihara, Akira Okada, Taiko Kusano, Kazuyoshi Ishigaki, Akira Shimizu, Hideki Takano

**Affiliations:** 1 Department of Nephrology, Tokyo Teishin Hospital, 2-14-23, Fujimi, Chiyoda-ku, 102-8798 Tokyo Japan; 2 Divison of Nephrology and Endocrinology, The University of Tokyo Graduate School of Medicine, 7-3-1, Hongo, Bunkyo-ku, 113-8655 Tokyo Japan; 3 Department of Allergy and Rheumatology, The University of Tokyo Graduate School of Medicine, 7-3-1, Hongo, Bunkyo-ku, 113-8655 Tokyo Japan; 4 Department of Analytic Human Pathology, Graduate School of Medicine, Nippon Medical School, 1-1-5, Sendagi, Bunkyo-ku, 113-8603 Tokyo Japan

**Keywords:** IgA vasculitis, Henoch-Schönlein purpura, Behçet’s disease, IgA nephritis, Renal biopsy, Pathergy reaction, HLA-B51

## Abstract

**Background:**

A pathergy reaction is defined as a hyperreactivity of the skin in response to minimal trauma, which is important in the diagnosis of Behçet’s disease (BD). However, a pathergy reaction may not be restricted to the skin, and little is known about whether an invasive medical procedure can induce the reaction. Here we present a pathergy reaction induced by renal biopsy, an invasive procedure.

**Case presentation:**

A 46-year-old man who was diagnosed with IgA vasculitis (IgAV) at the age of 38 was treated with prednisolone and mizoribine. However, complications such as common carotid arteritis or recurrent oral ulcer suggested the possibility of another pathophysiology. Later, increasing urine protein developed, suggesting disease aggravation. However, renal biopsy showed arteriosclerotic changes caused mainly by hypertension, negating exacerbation. After renal biopsy, his renal dysfunction and body temperature fluctuated, and detailed examinations revealed recurrent oral and genital ulcers and a folliculitis-like rash on his scrotum. Later, he complained of myodesopsia caused by hemorrhage in the ocular fundus due to occlusive vasculitis. Complete BD was diagnosed after development of the symptoms, and he was treated with prednisolone and colchicine.

**Conclusion:**

Co-occurrence of BD with IgAV is very rare and may be associated with immune disorders. Interestingly, a renal biopsy revealed BD, which was masked by the presence of IgAV, and elucidated the etiology of the unexplainable symptoms. To the best of our knowledge, this is the first report of renal pathergy. This case enlightens clinicians to the fact that not only a needle stimulation but also an invasive procedure can cause a pathergy reaction.

## Background

Behçet’s disease (BD) is characterized by recurrent oral and genital ulceration, ocular disease, and several systemic manifestations, including skin lesions, gastrointestinal involvement, neurologic disease, vascular disease, and arthritis [1]. BD can involve blood vessels of all sizes; hence, most of its clinical manifestations are caused by vasculitis [2]. In 1963, the first occurrence of proteinuria and hematuria was reported in 13 of 65 BD patients [3]. Thereafter, many reports showed renal involvement with BD [4, 5]. The association with IgA vasculitis (IgAV) and BD is not clear [6–9]; few reports have shown a pathergy reaction activated at other tissue sites [10], and we experienced a rare case of pathergy reaction induced by renal biopsy, an invasive procedure. To our knowledge, this is the first report of a BD patient who experienced a renal pathergy reaction.

## Case presentation

A 46-year-old male was first referred to a nephrologist at the age of 15 years due to proteinuria and hematuria. Renal biopsy revealed IgA nephritis (IgAN). At 38 years, his history of IgAN was complicated with abdominal pain and painful palpable purpura on his lower legs. A skin biopsy revealed leukocytoclastic vasculitis. Thus, he was diagnosed as having Henoch-Schönlein purpura (IgAV). On the other hand, some of his complications were unexplainable by the original disease alone, such as common carotid arteritis and recurrent oral ulcer. Although he was diagnosed with steroid-induced diabetes mellitus at the age of 40, it was under control with HbA1c levels approximately 7% on average; furthermore, multiple examinations by an ophthalmologist revealed no diabetic retinopathy. As a treatment for IgAV, oral prednisolone (PSL) was started at 50 mg/day, and his purpura and urine abnormalities resolved; PSL dose was gradually reduced.

**Fig. 1:**
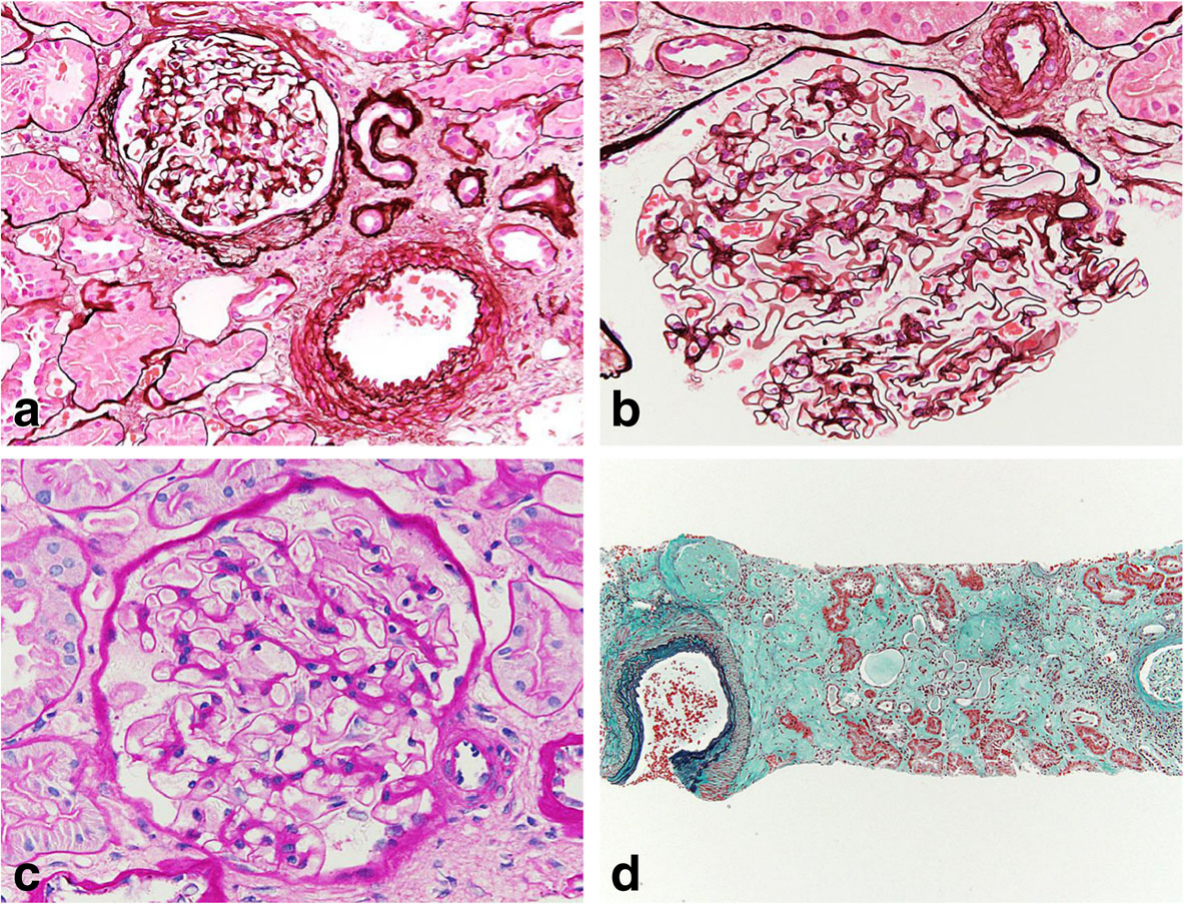
Light microscopic images of renal biopsy. **a** The biopsy highlighted 14 glomeruli, of which two were obsolescent, four were adherence lesions with fibrous crescents showing previous active IgA vasculitis (periodic acid-silver methenamine stain; original magnification, ×200). **b**, **c** Other glomeruli showed little hypertrophy and minor glomerular abnormalities (**b** periodic acid-silver methenamine stain; **c** Periodic acid-Schiff stain; original magnification, ×600). **d** Tubulointerstitial atrophy developed and showed an arteriosclerotic lesion with hypertension and diabetes (Masson trichrome stain; original magnification, ×40)

**Fig. 2:**
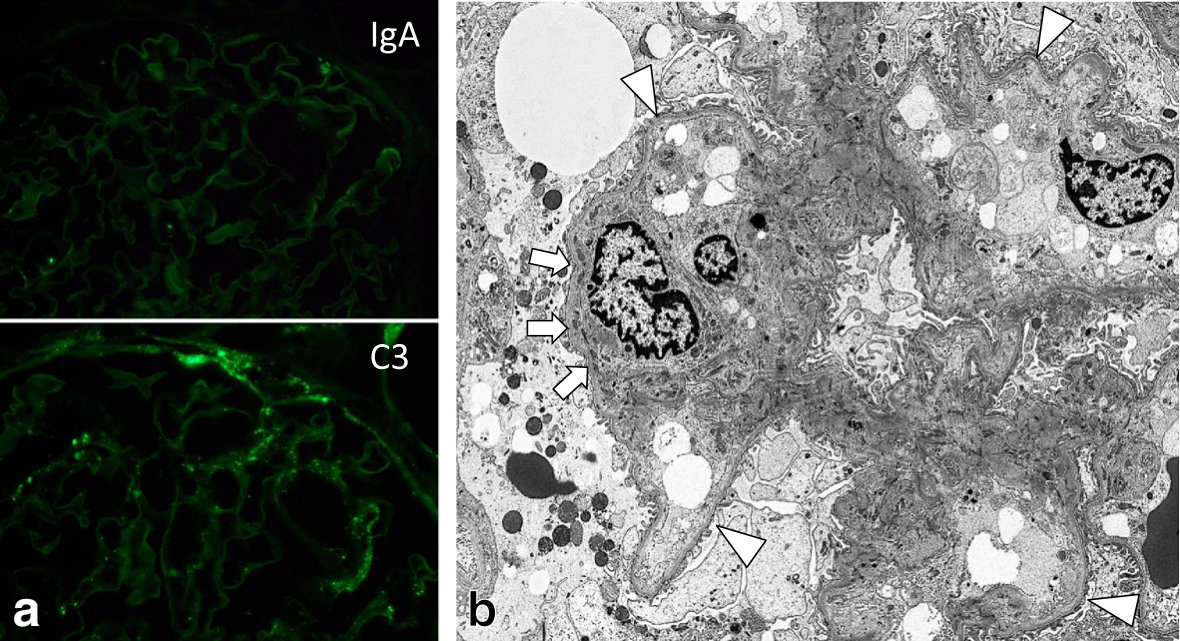
Immunofluorescence staining and electron microscopy of the renal biopsy. **a** Immunofluorescence staining for IgA and C3 showed negative staining in the glomeruli (original magnification, ×600). **b** Electron microscopy revealed small electron-dense deposits (*arrows*) under the basement membrane. In addition, diffuse thickening of glomerular basement membrane (GBM), which is known as an early ultrastructural characterization of diabetic nephropathy, was not detected (sites of GBM not affected pointed in *arrowheads*) (original magnification, ×4000)

**Fig. 3:**
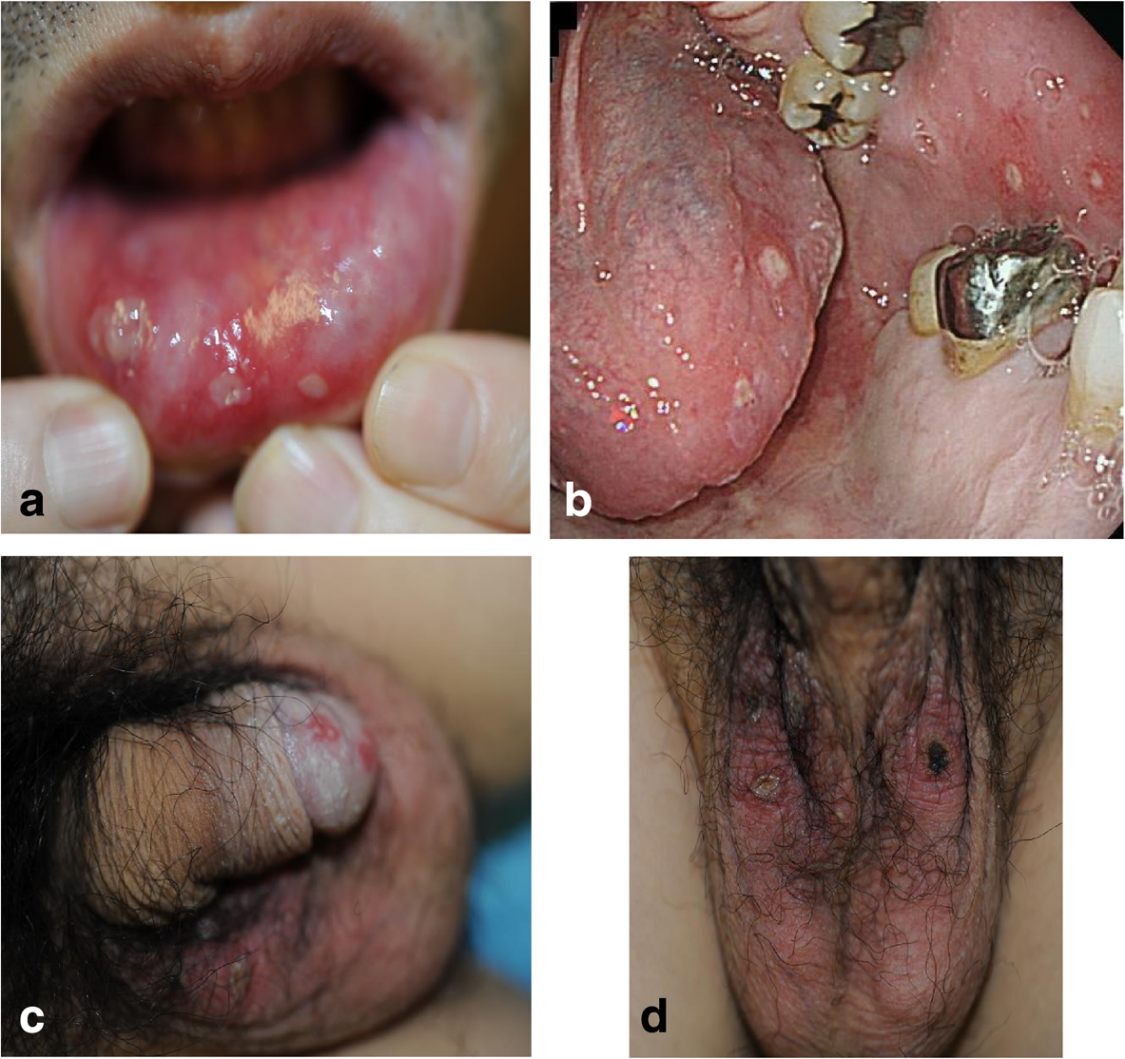
Physical examination. **a** Ulcers on the lower lip. **b** Ulcers on the tongue and buccal mucosa. **c** Genital ulcers. **d** Folliculitis-like rash on the scrotum

**Fig. 4:**
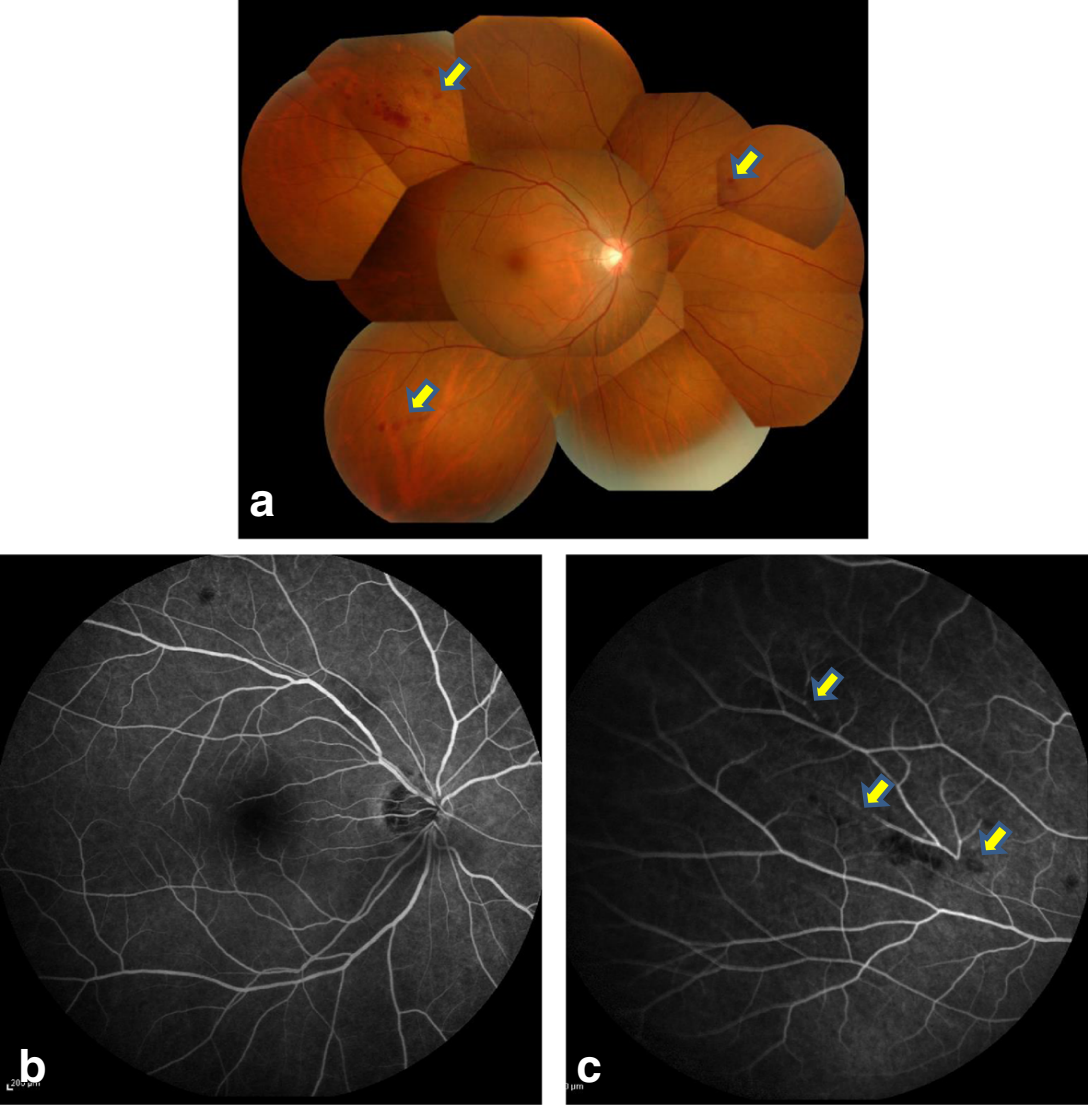
Ophthalmologic examination. **a** Fundus examination: hemorrhage in the peripheral retina (*arrows*). **b**, **c** Fluorescent fundus angiography. There was no microaneurysm seen in diabetic retinopathy. The area of retinal hemorrhage and local filling defect of the vein are indicated by *arrows*

IgAV relapsed at the age of 45 with increasing urine protein. Tonsillectomy plus triple methylprednisolone pulse therapy did not ameliorate the urine protein aggravation. Renal biopsy was performed and light microscopic analysis of the specimen revealed 14 glomeruli; of these, 2 were obsolescent, 4 were adherence lesions with fibrous crescents showing previous active IgAV, and the others exhibited only minor glomerular abnormalities. Immunofluorescence staining was negative for IgA or C3 in the glomeruli, and electron microscopy revealed no evidence of an early sign of diabetic nephropathy such as diffuse thickening of glomerular basement membrane. As a result, no exacerbation of the original disease and no onset of other specific kidney lesions, such as diabetic nephropathy, were observed (Figs. 1 and 2). After the biopsy, intermittent fever developed and the serum creatinine temporally changed from 0.68 to 2.26 mg/dl. In addition, a prolonged fever necessitated a detailed laboratory analysis (Table 1). Microbacterial and imaging inspections, including Ga-scintigraphy, could not detect any inflammatory focus; however, a physical examination revealed a folliculitis-like rash on his scrotum (Fig. 3). Ophthalmologic examination and gastroscopy and colonoscopy tests yielded negative results. At first, we diagnosed an incomplete type of BD, which was treated with PSL. The increased dose of PSL failed to relieve the fever and finger pain, and then, an ophthalmologic reexamination revealed hemorrhage in the ocular fundus (Fig. 4). In addition to the absence of microaneurysms, local filling defects of the vein were seen at the area of retinal hemorrhage, suggesting BD-associated vasculitis. After the diagnosis of complete-type of BD was made, the PSL dose was increased to 30 mg daily. Urinary protein levels subsequently improved along with amelioration of BD, which supported that diabetes had little contribution to the renal lesion, considering the absence of diabetic retinopathy and short history. Despite tapering of the PSL, no exacerbation was observed with the daily oral 8 mg of PSL and 1 mg of colchicine.

**Table 1:** Laboratory tests

**Blood test**		γ-glutamyl transferase	25 IU/l	anti-dsDNA	Negative
White blood cells	10,800/μl	Fasting blood glucose	117 mg/dl	MPO-ANCA	Negative
Hemoglobin	11.6 g/dl	Hemoglobin A1c	7.4%	PR3-ANCA	Negative
Platelets	38.3 × 10^4^/μl	APTT	23.9 s	anti-GBM	Negative
Sodium	137.1 mEq/L	PT-INR	1.05	HIV	Negative
Potassium	3.6 mEq/L	FDP	5.2 μg/ml	HCV antibody	Negative
Chloride	100.5 mEq/L	C-reactive protein	1.87 mg/dl	HBs antigen	Negative
Calcium	9.0 mg/dl	ESR	101 mm/h	TPHA	Negative
Phosphate	2.3 mg/dl	IgA	330 mg/dl	**Urine test**	
Creatinine	0.74 mg/dl	IgG	948 mg/dl	pH	6.0
Urea Nitrogen	7.6 mg/dl	IgM	87 mg/dl	Occult blood	Negative
Total protein	6.6 g/dl	IgE	48 IU/ml	Glucose	Negative
Albumin	3.2 g/dl	C3c	159 mg/dl	White blood cells	Negative
Lactate dehydrogenase	181 IU/l	C4	51 mg/dl	Urine protein	1.01 g/gCr
Aspartate aminotransferase	16 IU/l	CH50	83 U/ml	NAG	15.1 U/l
Alanine aminotransferase	25 IU/l	Anti-nuclear antibody	Negative	β2-MG	1102 ng/ml

## Conclusions

BD is a recurrent multisystem inflammatory disorder characterized by vasculitis and arteritis [1, 2] and involves blood vessels of all sizes [11–13]. Conversely, IgAV is defined as vasculitis with IgA1-dominant immune deposits; it affects small vessels, skin, and the gastrointestinal tract and causes arthritis [14]. In this case, IgAV caused abdominal pain and painful palpable purpura; however, the patient presented at the same time with bilateral common carotid arteritis on carotid ultrasonography. The latter is explained by coexistence of BD, which can affect large arteries. In addition, he also had recurrent oral ulcers before the diagnosis of IgAN, which also supports the coexistence of the two diseases. However, the activity of BD was suppressed by steroid therapy as an IgAV treatment but was coincidentally activated by a pathergy reaction, which was identified during a renal biopsy.

A pathergy reaction is defined as hyperreactivity of the skin in response to minimal trauma [15]. This is important in the diagnosis of BD and is assigned 1 point based on the International Criteria for BD, which classifies a patient scoring ≥4 points as having BD [16]. The pathergy reaction was not restricted to the skin [10]; it also may be associated with superficial thrombophlebitis induced by venipuncture [17, 18], an erythematous lesion on the conjunctiva after intravitreal corticosteroid injections [19], anastomotic ulcers after surgical treatment of intestinal lesions [20], and aneurysm after vascular surgery [21, 22]. We could not confirm a clear presentation of inflammation on the puncture site in our case; however, renal biopsy activated a pathergy reaction in the kidney, which is rich in blood vessels. We found that a renal biopsy itself can reveal another vasculitis complication.

Albeit rarely, BD can complicate IgAV or IgAN; only a limited number of articles have been reported [6–9, 23, 24]; thus, there may be something unusual about the coexistence. BD with renal involvement can present in certain human leukocyte antigens (HLA) differently from BD without renal involvement. Interestingly, previous studies have reported that only one of seven BD patients with renal involvement had HLA-B51 [25], which is associated with an increased risk of developing BD [26], while HLA-A2, A11, and particularly B35 are associated with an increased risk of IgAV [27]. The HLA types of blood lymphocytes in our patient were A2, B35, B48, DR8, and DR12, which suggests a high possibility of developing IgAV; however, our patient did not have HLA-B51. A negative finding for HLA-B51, as observed in our case, also may be a characteristic in BD patients with renal involvement, and thus, HLA-B51 may not be helpful in diagnosing BD with renal lesions.

BD can be diagnosed only by detailed physical examination; however, this is difficult when it is not suspected or masked by presentation of other forms of vasculitis or during immunosuppression treatment. Surprisingly, a renal biopsy disclosed “masked” BD by inducing a renal pathergy reaction; therefore, the etiology of common carotid arteritis and recurrent oral ulcer was unveiled because IgAV usually does not affect middle or large sized arteries. If it had not been for the renal biopsy, we may have failed to diagnose or treat the patient’s coexisting BD. This case suggested that we should perform focused history-taking and physical examinations when some signs and symptoms in a patient cannot be explained fully by one form of vasculitis, for example, IgAV.

## Abbreviations

ANCA: Anti-neutrophil cytoplasmic antibody
APTT: Activated partial thromboplastin time
BD: Behçet’s disease
ESR: Blood sedimentation rate
FDP: Fibrin degradation products
HIV: Human immunodeficiency virus
HLA: Human leukocyte antigens
HSP: Henoch-Schönlein purpura
IgAN: IgA nephritis
IgAV: IgA vasculitis
MPO: Myeloperoxidase
NAG: N-acetyl-D-glucosamine
PR3: Proteinase-3
PSL: Prednisolone
PT-INR: Prothrombin time-international normalized ratio
RH: Retinal hemorrhage
TPHA: Treponema pallidum hemagglutination test
β2-MG: β2-microglobulin
